# Increasing incidence of anogenital warts with an urban–rural divide among males in Manitoba, Canada, 1990–2011

**DOI:** 10.1186/s12889-016-2885-4

**Published:** 2016-03-03

**Authors:** Laura H. Thompson, Zoann Nugent, James F. Blanchard, Carla Ens, Bo Nancy Yu

**Affiliations:** Epidemiology and Surveillance Unit, Public Health and Primary Health Care Division, Manitoba Health, Healthy Living & Seniors, Winnipeg, R3B 3 M9 Manitoba Canada; Centre for Global Public Health, University of Manitoba, Winnipeg, Canada; Department of Community Health Sciences, University of Manitoba, Winnipeg, Canada

**Keywords:** Anogenital warts, Anogenital conditions, Human papillomavirus, Epidemiology, Manitoba

## Abstract

**Background:**

Anogenital warts (AGW) are caused by the most common sexually transmitted infection, human papillomavirus. The objective of this study was to examine AGW incidence from 1990 to 2011 by sex, age, income quintile, and residential area category (urban/rural). The study period included the initiation of school-based HPV vaccination for girls in the sixth grade, which began in 2008. The data presented in this paper may also be useful for establishing baseline rates of AGW incidence which may be used to evaluate the success of the school-based HPV immunization program.

**Methods:**

Cases of anogenital warts were identified using Manitoba’s administrative databases of Physician Claims and Hospital Discharge Abstracts. Annual age-standardized incidence in Manitoba from 1990 to 2011 was calculated. Incident AGW rates were compared by sex, age group, residential area category (urban/rural), and household income quintile using logistic regression. Joinpoint regression analyses were used to evaluate the time trends of AGW.

**Results:**

Prior to 2000, AGW incidence was higher among females than males. However, from 2000 to 2011 the incidence was higher among males and increased steadily over time. AGW incidence tended to peak in younger age groups among females compared to males. Females and males living in urban areas had nearly twice the odds of AGW occurrence compared to those in rural areas.

**Conclusions:**

There is a need for education about AGW in male population. The upcoming initiation of HPV vaccination among boys may reduce the incidence and should be evaluated.

**Electronic supplementary material:**

The online version of this article (doi:10.1186/s12889-016-2885-4) contains supplementary material, which is available to authorized users.

## Key messages

- Anogenital warts incidence was higher among females prior to 2000, but since then has been higher among males than females and has increased steadily.
- AGW incidence was higher among males at all age groups except 15–19; by age 25 the incidence was 1.5 to 2 times higher than females.
- Both females and males living in urban areas had twice the odds of AGW occurrence compared to those in rural areas.

## Background

Anogenital warts (AGW) are caused by the most common sexually transmitted infection, human papillomavirus (HPV) [1]. Asymptomatic cervical HPV infection may be detected among 5 % to 40 % of females of reproductive age [2]. Over time, an individual may be infected with different HPV subtypes and may clear some HPV infections naturally [1, 2]. It is estimated that 90 % of AGW are caused by HPV type 6 and HPV type 11 [3, 4].

AGW may be visible as single or multiple flesh-coloured bumps in the anogenital region. Whether treated or not, AGW are highly infectious and recurrent [5] and are associated with relatively high treatment costs. A study of the costs associated with AGW in British Columbia from 1998 to 2006 found that on average the treatment of one episode of AGW cost $176 for males and $207 for females [6]. A recent review based on 32 articles published globally on the topic of AGW incidence from 2001 to 2012 found that the incidence of AGW ranged from 160 to 289 per 100,000, and peaked before the age of 24 among females and between 25 and 29 years of age among males [7]. Only one other article described AGW incidence in Manitoba, and it included data up to 2004 [8]. In the year that AGW incidence was last reported, 2004, the incidence of AGW among females was 120 cases per 100,000 persons and among males it was 154 cases per 100,000 persons. From 1985 to 1999, the incidence of AGW in Manitoba was higher among females than males, and showed a downward trend for both from 1992 to 1999 [8]. The incidence in almost all age groups continued to increase among males but decreased among females from 2000 to 2004, and beginning in the year 2000 the incidence has been higher among males than females [8]. It is not known whether the increasing trend of AGW in men will continue. It is also lack information on the characteristics of this increasing trend of AGW in men.

An HPV vaccine (Gardasil), which provides protection against HPV types 6, 11, 16, and 18, has been available free-of-charge to all females in Grade 6 in Manitoba since September 2008. Since November 2012, all females between 9 and 26 years of age with an increased risk of HPV infection (determined by a health care provider) are also eligible to receive the HPV vaccine [9]. Starting in September 2016, the Manitoba government will further expand the HPV vaccination program to include males in Grade 6 or born on or after January 1^st^ 2005 [10]. Population-based baseline data of the incidence of AGWs in females and males is useful for the evaluation of the success of these vaccination initiatives.

The objective of this study was to examine AGW incidence from 1990 to 2011 by sex, age, income quintile, and residential area category (urban/rural), determining whether the increase in AGW rate among males detected in 2004 continued. The study period included the initiation of school-based HPV vaccination for girls (which began in 2008) and but not boys (which will begin in 2016) in the sixth grade. The data presented in this paper will be useful for establishing baseline rates of AGW incidence which may be used to evaluate the success of the school-based HPV immunization program.

## Methods

### Data sources

Annual age-standardized incidence of AGW in Manitoba from 1990 to 2011 was calculated by sex, age group, income quintiles, and geographic residential area (urban or rural). The two major cities in Manitoba are Winnipeg, Manitoba’s biggest urban centre (estimated population 672,000) and Brandon (estimated population 46,000 population) [11]. These two cities are grouped as the “urban” residential category and include 60 % of Manitoba’s population. The rest of Manitoba is the “rural” residential category.

Data was obtained from the centralized Manitoba Health, Healthy Living and Seniors databases, including: the Manitoba Population Registry, Physician Claims, and Hospital Discharge Abstracts. The Manitoba Population Registry was used to identify all Manitobans who were eligible for provincial health insurance benefits. Physician Claims (including shadow billing) and Hospital Discharge Abstracts were used to identify cases of AGW. Specifically, the de-identified, individual, line-level data from physician and hospital claims (which captured AGW cases diagnosed and treated in Manitoba) were utilized. Shadow billings are claims submitted to the provincial government, by physicians who are not fee-for-service, for administrative purposes only (i.e. as a record of services provided).

Manitoba population data for June 30^th^ of each year were provided by the Health Information Management Branch of Manitoba Health, Healthy Living and Seniors.

### Standard protocol approvals, registrations, and patient consents

The University of Manitoba Health Research Ethics Board approved the study. As a public health surveillance activity, a notification by the Manitoba Health Information Privacy Committee for analyzing administrative data was obtained. This study involved the use of anonymous provincial health insurance administrative data and did not involve any direct patient contact. Consent from patients was not required.

### Case definition

AGW cases were identified based on the procedure codes specific to condyloma (Additional file 1: Table S1 and Table S4, S5) from the medical claims data. The condyloma procedure codes are analogous to the Current Procedure Terminology codes used in the United States. The diagnosis and procedure codes, International Classification of Diseases [(ICD), Ninth Revision and 10^th^ Revision (ICD 9/10)] recorded in the hospital records were used to identify the AGW cases in hospital settings (Additional file 1: Table S2 and Table S3). Each incident AGW occurrence (case) was defined as one “episode of care” to distinguish it from subsequent occurrences (see below). AGW-specific condyloma tariff (physician billing) and procedure (Hospital Discharge Abstracts) codes listed in two administrative databases from 1990 to 2011 were used to identify AGW cases.

For those AGW that were treated during a hospital stay and did not generate a record in the physician claims database, a combination of ICD (9/10) diagnosis codes and procedure codes were used to identify the AGW cases.

The case definition used in this study is consistent with the case definition used for previous studies of AGW [8].

#### Episode of care of anogenital warts and incident anogenital warts cases

An “episode of care” includes all AGW diagnoses and AGW-related billing which occurred within a twelve month period of time. If another AGW claim occurred more than twelve months after a previous AGW episode of care, it was considered a new AGW episode (i.e. a new AGW case).

Episodes of care were identified using the hospital and physician claims databases. If any of the below occurred more than twelve months after a previous AGW episode of care, it is considered a new AGW episode:

- an AGW-related tariff code, OR
- an AGW ICD-9/10 code, OR
- an AGW-related procedure code followed by another claim within two weeks that had an AGW-related ICD code (ICD9 = 078, Other disease due to viruses and Chlamydia).

If any of the above occurred within twelve months of a previous episode of care, then it is considered as part of the same AGW episode of care.

The diagnostic date for a new AGW case was defined as the earliest date listed in hospital or physician claims for that particular episode of care. If a person died or moved out of province within the twelve month period of the AGW episode of care, the coverage cancellation date, recorded in the Manitoba Population Registry, was used as the end of that episode of care.

### Statistical analysis

Age-standardized incidence was calculated using incident cases as the numerator and mid-year age-specific populations as the denominator. All incidence rates are reported as cases per 100,000 persons in the Manitoba population. Ninety-five percent confidence intervals (95 % CI) of rates were calculated, and formal statistical testing for incidence rate ratios was undertaken using Poisson regression, with the natural log of the population as an offset variable. As personal income data is not available in the administrative database, we linked the median household income at the neighbourhood-level (forward sortation area; ie. first 3 digits of postal code) from Canadian Census data in 2001 to the residential area of patients recorded in the databases as a proxy for socioeconomic status [12–14]. Median household income was grouped into quintiles, less than CAD $17,414 as the lowest quintile (Q1), CAD$17,415–20,411 as second quintile (Q2), CAD$20,412–24,422 as the third quintile (Q3), CAD$24,423–27,848 as the fourth quintile (Q4), and CAD$27,845–35,122 as the highest quintile (Q5). Based on the residential postal codes, data were grouped as rural or urban category. The two major urban centers (Winnipeg and Brandon) were categorized as urban and the rest of the province was categorized as rural. Cases with a missing or unknown postal code or originating from out of province were excluded from the region-specific analyses. Household income quintile categories were obtained by linking the forward sortation area code (first three digits of the postal code) to Canadian census data [15].

Incident AGW rates were compared by sex, age group (0–14, 15–19, 20–24, 25–29, 30–39, 40–49, 50–59, and 60+ years), residential area category (urban versus rural), five-year period, household income quintile using logistic regression. Results of regression models are summarized as adjusted odds ratios with 95 % confidence intervals (CI). These analyses were conducted using SAS v9.4 (SAS Institute, Cary, NC).

Historical cohort analyses were performed to assess the rates and time trends of AGW in females and males. Joinpoint regression analyses [16–18], developed by SEER (Surveillance Epidemiology and End Results, National Cancer Institute, United States), were used to evaluate the changes in time trends of AGW in females and males, and further by urban and rural residential areas.

This study was approved by the Health Research Ethics Board at University of Manitoba.

## Results

### Overall time trends of anogenital warts incidence in Manitoba

**Fig. 1:**
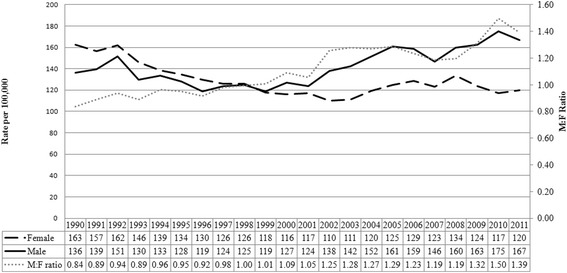
Age-Standardized Incidence (per 100,000 persons) of AGW in Manitoba, 1990 – 2011ᅟ

There were 31,510 AGW cases identifiable in the administrative databases in Manitoba from 1990 to 2011; 15,208 among females and 16,302 among males. The age-standardized incidence of AGW was higher among females compared to males until 1999 (Fig. 1). However, this trend has reversed since 2000 with AGW incidence higher among males than females. Among males, the age-standardized AGW incidence increased 31 % from 124 (95 % CI 114–134) cases per 100,000 persons in 2001 to 175 (95 % CI 164–187) cases per 100,000 persons in 2010 (Fig. 1). Consequently, the male to female incidence rate ratio steadily increased from 1.05 (95 % CI 0.94–1.18) in 2001 to 1.50 (95 % CI 1.35–1.65) in 2010 and 1.39 (95 % CI 1.26–1.54) in 2011. Time trend analysis revealed two distinctive time periods and changing rates of AGW in males and females, respectively. The average annual change in AGW incidence was–3.35 % among females from 1990 to 2000 (*p* < 0.001), and incidence did not change from 2000 to 2011 (annual percent change 0.79, *p* = 0.09). The average annual change of AGW incidence in males was −1.86 % from 1990 to 1998 (*p* = 0.016), and 2.80 % from 1998 to 2011 (*p* < 0.001).

Compared to the earliest time period (1990–1994), the odds of AGW occurrence among females were consistently low (between 0.75 and 0.83) after adjusting for age group, geographic residential area category, and income quintile (Table 1). Starting in 2005, males had a greater odds of AGW compared to the time period 1990–1994.

**Table 1:** Associations of AGW Incidence (per 100,000) and Demographic Factors in Manitoba, 1990–2011

Effect	Female and male combined		Female		Male	
	Incidence (95 % CI)	Odds ratio (95 % CI)	Incidence (95 % CI)	Odds ratio (95 % CI)	Incidence (95 % CI)	Odds ratio (95 % CI)
All	123 (122–125)					
Female	117 (115–119)	Ref	117 (115–119)			
Male	129 (127–131)	1.08 (1.05–1.10)*			129 (127–131)	
Age 0–14	10 (9–11)	0.02 (0.02–0.02)*	9 (8–11)	0.02 (0.02–0.02)*	10 (9–12)	0.02 (0.02–0.03)*
Age 15–19	212 (205–218)	0.46 (0.44–0.48)*	326 (314–338)	0.65 (0.62–0.69)*	103 (96–109)	0.24 (0.23–0.26)*
Age 20–24	480 (470–490)	Ref	520 (505–536)	Ref	440 (426–453)	Ref
Age 25–29	337 (329–346)	0.69 (0.67–0.71)*	271 (261–282)	0.51 (0.49–0.54)*	403 (390–416)	0.90 (0.86–0.94)*
Age 30–39	177 (173–181)	0.37 (0.35–0.38)*	136 (131–141)	0.26 (0.25–0.27)	218 (211–225)	0.49 (0.47–0.51)*
Age 40–49	93 (90–96)	0.19 (0.19–0.20)*	79 (75–83)	0.15 (0.14–0.16)*	106 (102–111)	0.24 (0.23–0.25)*
Age 50–59	53 (50–55)	0.11 (0.10–0.12)*	46 (43–50)	0.09 (0.08–0.10)*	59 (55–63)	0.13 (0.12–0.14)*
Age 60+	22 (21–23)	0.05 (0.04–0.05)*	15 (14–17)	0.03 (0.03–0.03)*	30 (28–33)	0.07 (0.06–0.08)*
1990–1994	140 (137–144)	Ref	146 (142–151)	Ref	134 (130–139)	Ref
1995–1999	114 (111–117)	0.86 (0.83–0.89)*	115 (111–119)	0.83 (0.79–0.87)*	113 (109–117)	0.89 (0.85–0.93)*
2000–2004	112 (109–114)	0.86 (0.84–0.89)*	102 (98–105)	0.75 (0.72–0.79)*	122 (118–126)	0.99 (0.95–1.04)
2005–2009	125 (122–128)	0.97 (0.94–1.00)	112 (109–116)	0.83 (0.79–0.86)*	138 (134–142)	1.14 (1.09–1.19)*
2010–2011	128 (123–132)	0.99 (0.95–1.03)	105 (99–111)	0.77 (0.72–0.82)*	151 (144–158)	1.24 (1.17–1.31)*
Rural	74 (72–75)	Ref	75 (73–77)	Ref	73 (70–75)	Ref
Urban	155 (153–157)	1.84 (1.79–1.90)*	143 (141–146)	1.75 (1.67–1.83)*	167 (164–170)	1.93 (1.85–2.01)*
Income quintile 1	83 (81–85)	Ref	84 (81–87)	Ref	82 (79–86)	Ref
Income quintile 2	113 (110–115)	1.20 (1.15–1.24)*	107 (103–110)	1.16 (1.10–1.22)*	118 (114–122)	1.24 (1.18–1.31)*
Income quintile 3	139 (136–142)	1.15 (1.10–1.19)*	133 (129–137)	1.13 (1.07–1.20)*	146 (142–150)	1.17 (1.11–1.24)*
Income quintile 4	166 (162–170)	1.38 (1.32–1.43)*	153 (147–158)	1.32 (1.24–1.40)*	179 (173–186)	1.45 (1.37–1.54)*
Income quintile 5	139 (134–144)	1.14 (1.09–1.20)*	124 (118–131)	1.07 (1.00–1.15)*	154 (147–162)	1.24 (1.16–1.33)*

Note: *p<0.0001

### Incidence of anogenital warts by Age group and Sex in Manitoba

**Fig. 2:**
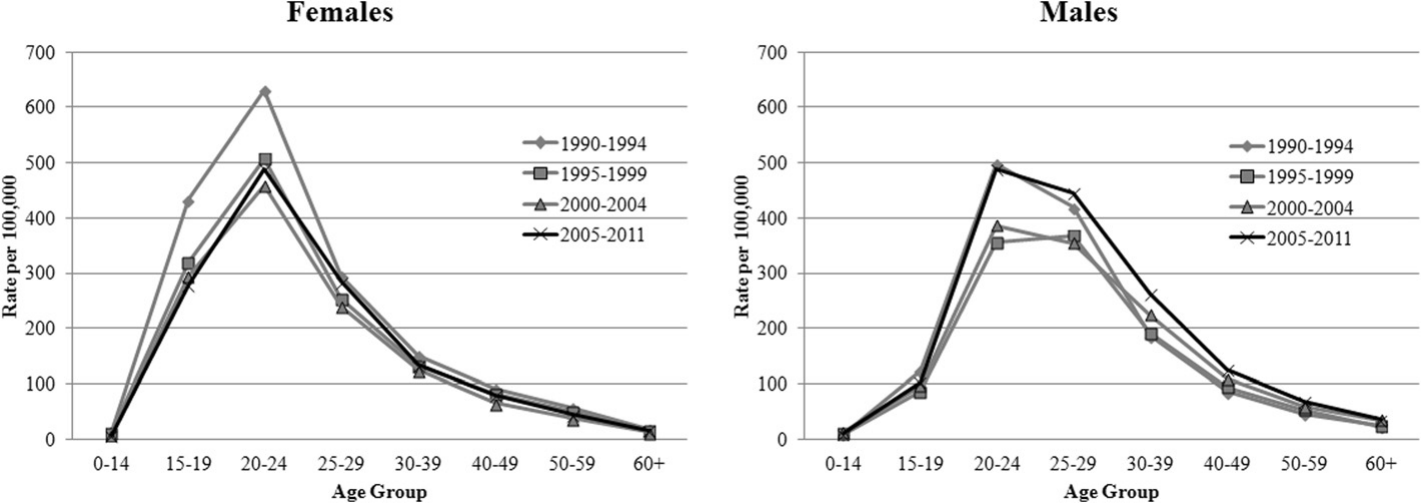
Sex Stratified Age-Specific 5-year Average AGW Incidence (per 100,000 persons) in Manitoba, 1990 – 2011

Among females, the incidence of AGW has been consistently higher among those aged 15–19, 20–24, and 25–29 years while among males it has been consistently highest in the 20–24, 25–29, and 30–39 age groups (Fig. 2). The highest incidence of AGW occurred among those aged 20–24 in both males and females (Table 1 and Fig. 2). As illustrated in Fig. 2, AGW incidence was highest among females aged 15–19 and 20–24 and males aged 20–24 during the period of 1990 to 1994 and among females in this age group the incidence has been substantially lower since then. Among males in this age group, the same high incidence was reached again in 2005–2011.

### Urban effects on the incidence of anogenital warts

**Fig. 3:**
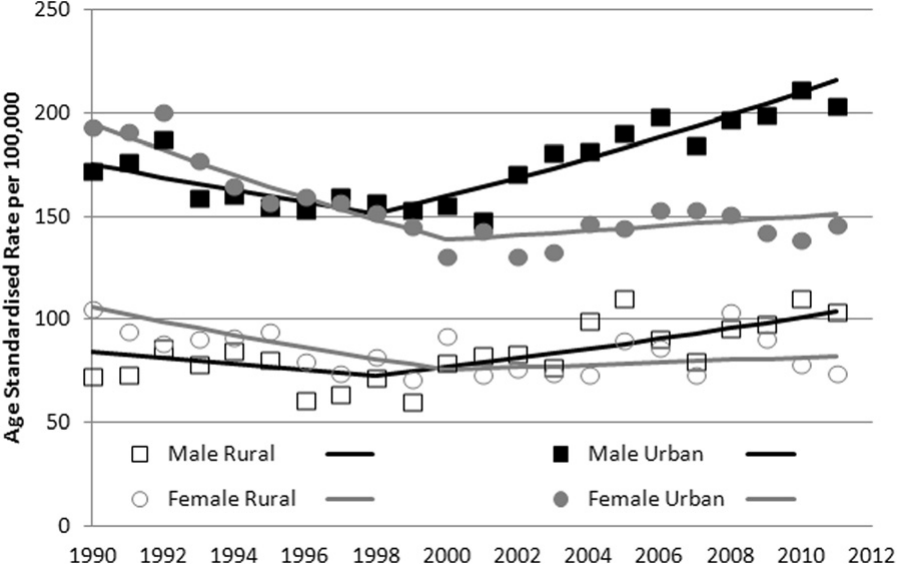
The Trends of Age-Standardized Incidence (per 100,000 persons) of AGW by Sex and Geographic Residential Area in Manitoba, 1990 - 2011

AGW incidence was higher in urban areas for both females and males (Fig. 3). The trend lines of AGW incidence were parallel for urban and rural for both females and males. After adjusting for age group, 5-year time periods, and income quintile, both urban females and males had an almost 2-fold greater odds of AGW compared to their counterparts living in rural areas (Table 1). Multivariable logistic regression analysis for the time periods of 1990–1999 and 2000–2011 separately revealed two trend changes for males and females. From 1990 to 1999, males had 20 % lower odds of AGW than females (OR = 0.80, 95 % CI 0.75–0.86). However, from 2000 to 2011, males had 7 % greater odds of AGW infection (OR = 1.07, 95 % CI 1.01–1.14) after adjusting for the interaction of residential area and age.

A substantial increasing trend of AGW incidence occurred among both urban and rural males after 1998 (Fig. 3). However, AGW rates in urban and rural females showed a steady downward trend since 1990, at an average downward change of 3.35 % (*p* < 0.001) per year, and stabilized from 2000 to 2011.

## Discussion

This study provided population-based descriptive epidemiologic information about AGW in Manitoba from 1990 to 2011. The incidence was found to be higher among females than males prior to 2000, which is consistent with previously published data [8]. Since 2000, the incidence of AGW has been higher among males than females and has increased steadily, with the incidence among females remaining steady and therefore the gap in incidence between females and males widening over time. Consequently, the male to female incidence rate ratio also steadily increased as the incidence of AGW among males grew to 50 % higher than females by 2010. A similar pattern of increasing AGW incidence among males was observed in Quebec, where it increased from 83 per 100,000 in 1998 to 103 per 100,000 in 2007 [19]. This is consistent with the sex-specific results of 32 studies conducted globally during the period of 2001 to 2012, whereby the median incidence among males (137 per 100,000 per year) was indeed higher than that among females (120.5 per 100,000 per year) [7]. The changing sex ratio in sexually transmitted diseases has been suggested as a surrogate marker of changes in sexual behaviors among men who have sex with men (MSM) [20]. The rising trends of AGW in males is concurrent with the increasing trends of gonorrhea and syphilis in males in Canada [21]. The resurging syphilis in males is largely attributed to the high-risk sexual practices among some MSM, such as using club drugs, multiple sexual partners, and larger sexual networks formed using the internet [22–27]. Therefore, changes in the sexual practices among MSM may also be an underlying factor for the rising AGW incidence among males in Manitoba. However, because sexual behavior and sexual orientation data are not available in the health insurance administrative databases, this study cannot assess the possible relationship between rising AGW incidence and trends in sexual behaviours.

AGW incidence tended to peak in younger age groups among females compared to males. The male to female incidence ratio indicated an often much higher incidence among males at all age groups except 15–19, where the incidence among males was only 40 % that of females. However, by the age of 25 the incidence of AGW among males was one-and-a-half to two times the incidence among females. These results are consistent with a review of 32 articles published globally on the topic of AGW incidence from 2001 to 2012, which found that the incidence of AGW peaked before the age of 24 among females and between 25 and 29 years of age among males [7.] However, the incidence observed in Manitoba over this time period among those aged 20–24 (520 and 440 cases per 100,000 among females and males, respectively) was notably higher than that observed elsewhere in Canada for the same age group, which ranged from 338 to 342 per 100,000 among females and 270 to 300 among males in BC and Quebec, respectively, for a similar time period [6, 28].

Both females and males living in urban areas had almost twice the odds of AGW occurrence compared to those in rural areas. The same temporal trend in which the incidence among males exceeded that of females and increased over time beginning in 2000 was observed in both urban and rural areas. The higher incidence of AGW in urban areas compared to rural areas may relate to greater MSM populations in urban areas [29] and differences in sexual mixing patterns, which are known to be associated with HIV, gonorrhea, and chlamydia risk [30–32]. It has been reported that for HPV type 6 and HPV type 11, which are most commonly associated with AGW, the prevalence among men who have sex with men (MSM) was very high (39.2–53.2 %) [33]. The higher AGW incidence and increasing trend that we observed among urban males could be partially related to high risk sexual behaviours in urban MSM populations [33]. It is also possible that individuals in urban areas are more likely to seek healthcare for AGW, and therefore were identifiable as AGW cases using the provincial administrative databases. Similar to our results, a study of AGW incidence in Germany from 2005–2006 revealed a higher incidence in urban areas compared to other regions [34]. To our knowledge these are the only reports of an observed urban–rural divide in AGW incidence. Further research is needed to elucidate possible explanations for the observed urban–rural differences in AGW incidence.

An online survey conducted across Canada in 2011 found that on average only half of the respondents believe AGW to be a serious STI, only one-quarter knew that it is associated with HPV, and 57 % were either not sure whether a condom would prevent transmission or believed that it would [28]. Other studies have indicated not only a lack of perceived risk for AGW among men who have sex with men in North America but also a lack of knowledge about the etiologic cause of AGW and the link between HPV infection and anal cancer [35, 36]. However, once informed of the role of HPV, hypothetical acceptance of HPV vaccine was common [35, 36]. HPV has been framed as a female problem, with emphasis on its etiologic role in cervical cancer and the targeting of vaccination programs towards females. This may partially explain the lack of awareness and increasing incidence among males since 2000. Education about HPV, its prevention, and the diseases it may cause are needed among both female and male populations. It has been recommended that HPV vaccination for boys could be an effective and cost-saving strategy for oropharyngeal cancer [37] and AGW [38]. An upcoming HPV immunization program among boys in Grade 6 in Manitoba will offer an important approach to reducing the high incidence of AGW among males. Research of the cost-effectiveness of the HPV vaccine in Manitoba and an evaluation of the acceptability and effectiveness of the Grade 6 vaccination program in reducing AGW incidence would provide useful information for programs.

The data presented in this report is subject to a number of limitations. It likely underestimates the actual occurrence of AGW in Manitoba as it relies on diagnosed cases and does not include those cases for which treatment was not sought. Further, incomplete shadow billing by physicians who are not fee-for-service and the exclusion of emergency department data may also result in an underestimate. The data included in this study were obtained from existing administrative databases compiled for other purposes and subject to the coding practices used over time. Although the accuracy of ICD codes and completeness of the diagnoses captured in hospital discharge records have been questioned [39, 40], the overall quality of the administrative databases has been demonstrated [41, 42]. Cases were based on clinical observations and not confirmed by laboratory results, though AGW clinical diagnoses have been found to correlate well with histological results [43].

Strengths of this population-based study include the fact that it is free from selection bias and the limitations of self-report data. Results of this study are generalizable to other similar populations with a similar medical system.

## Conclusions

Since 2000 the incidence of AGW among males in Manitoba has been increasing and is particularly high among those residing in urban areas. This observation is consistent with the increasing incidence of syphilis and gonorrhea among males in Manitoba and may be related to increasing sexual risk behaviours among some urban MSM populations. Lower AGW rates in rural males may be related to lower risk behaviours or healthcare seeking behavior if fewer seek healthcare, resulting in a smaller proportion of AGW cases being diagnosed compared to their urban counterparts. Moreover, HPV has been framed largely as a female problem, which may partially explain the lack of awareness and increasing incidence among males. More sexual health education may help patients overcome the stigma of early diagnosis of sexually transmitted infections and improve sexual health in general. There is a need for education about HPV, its prevention, and the diseases it may cause among both female and male populations. Also, effectiveness studies of the HPV vaccination program among Grade 6 males in Manitoba, to begin in 2016, will generate useful evidence about the importance of targeting prevention activities towards males.

## Additional file

Additional file 1: Table S1. Tariff codes used to identify a person with AGW in the medical claims. **Table S2.** ICD-9/10 diagnosis codes for anogenital warts. **Table S3.** ICD 9 procedure codes used to assist in the identification of a person with anogenital warts. **Table S4.** ICD 10 procedure codes used to assist in the identification of a person with anogenital warts. **Table S5.** Tariff codes used to assist in the identification of a person with anogenital warts. (DOCX 46 kb)
