# Supplementary material for: Increasing incidence of anogenital warts with an urban–rural divide among males in Manitoba, Canada, 1990–2011
Source: BMC Public Health. 2016 Mar 3;16:219. doi: 10.1186/s12889-016-2885-4 (PMC4778275; doi:10.1186/s12889-016-2885-4)
Supplement: Additional file 1: Table S1. — Tariff codes used to identify a person with AGW in the medical claims. Table S2. ICD-9/10 diagnosis codes for anogenital warts. Table S3. ICD 9 procedure codes used to assist in the identification of a person with anogenital warts. Table S4. ICD 10 procedure codes used to assist in the identification of a person with anogenital warts. Table S5. Tariff codes used to assist in the identification of a person with anogenital warts. (DOCX 46 kb) [file 12889_2016_2885_MOESM1_ESM.docx]

Supplemental Tables: Tariff Codes and ICD9/10 Codes for AGW

Table A. Tariff codes used to identify a person with AGW in the medical claims

| Code | Description |
| --- | --- |
| 3372 | Anus, condyloma, single or multiple, internal or external, destruction, in hospital |
| 3433 | Anus, condyloma, external, electrodessication, initial, per sitting |
| 3434 | Anus, condyloma, external, electrodessication, subsequent, per sitting |
| 4120 | Penis, penile skin lesion, including warts, local excision or fulguration, per sitting |
| 4412 | Vulva, condylomata excision or destruction any method less than 10 warts up to 25% of vulva |
| 4413 | Vulva, condylomata excision or destruction any method 10 or more warts more than 25% of vulva |
| 4415 | Vagina, condylomata excision or destruction any method less than 5 warts up to 25% of vagina |
| 4416 | Vagina, condylomata excision or destruction any method 5 or more warts more than 25% of vagina |
| 4422 | Vulva, condyloma accuminata local excision, fulguration, chemical application or injection or other treatment, per sitting |
| 4427 | Vulva, condyloma accuminata, extensive removal under general anaesthesia |
| 4430 | Vulva, condylomata excision or destruction any method less than 10 warts up to 25% of vulva |
| 4432 | Vulva, condylomata excision or destruction any method 10 or more warts more than 25% of vulva |
| 4472 | Vagina, condylomata excision or destruction any method less than 10 warts up to 25% of vagina |
| 4475 | Vagina, condylomata excision or destruction any method 10 or more warts more than 25% of vagina |

Table B. ICD-9/10 diagnosis codes for anogenital warts

| **Database date** | **ICD codes** | **Procedure Codes** |
| --- | --- | --- |
| Before April 1994 | 078.1 | Table B2.2 |
| April 1994 – March 2004 | 078.11, or 078.10, or 078.19 | Table B2.2 |
| After March 2004 | A630 or B07 | Table B2.3 |

Table C. ICD 9 procedure codes used to assist in the identification of a person with anogenital warts

| **Code** | **Description** |
| --- | --- |
| 48.82 | Excision of perirectal tissue |
| 49.04 | Other excision of perianal tissue |
| 49.3 | Local excision or destruction of other lesion or tissue of anus |
| 49.31 | Endoscopic excision or destruction of lesion or tissue of anus |
| 49.39 | Other local excision or destruction of other lesion or tissue of anus |
| 58.3 | Excision or destruction of lesion or tissue of urethra |
| 58.31 | Endoscopic excision or destruction of lesion or tissue of urethra |
| 58.39 | Other local excision or destruction of lesion or tissue of urethra |
| 61.3 | Excision or destruction of lesion or tissue of scrotum |
| 64.2 | Local excision or destruction of lesion of penis |
| 67.32 | Destruction of lesion of cervix by cauterization – electroconization of cervix |
| 67.33 | Destruction of lesion of cervix by cryosurgery – cryoconization of cervix |
| 67.39 | Other excision or destruction of lesion or tissue of cervix |
| 70.33 | Excision or destruction of lesion of vagina |
| 71.3 | Local excision or destruction of vulva and perineum |

Table D. ICD 10 procedure codes used to assist in the identification of a person with anogenital warts

| **Code** | **Description** |
| --- | --- |
| 1RS59CAGX | Destruction vagina using per orifice approach and device NEC |
| 1NT59CAGX | Destruction anus using per orifice approach and device NEC |
| 1PQ59LAGX | Destruction urethra using open approach and device NEC |
| 1RW59JAGX | Destruction vulva using external approach and device NEC |
| 1RW59JAX7 | Destruction vulva chemocautery agent |
| 1RY87LA | Excision, partial perineum |

Table D. Tariff codes used to assist in the identification of a person with anogenital warts.

| **Code** | **Description** |
| --- | --- |
| 0253 | Excision & simple closure – single lesion, any location |
| 0254 | Excision & simple closure – each additional lesion to a maximum of four |
| 0255 | Excision & closure – multiple lesions, extensive |
| 0397 | Laser vaporization, other than face, one lesion |
| 0398 | Laser vaporization, other than face, two lesions |
| 0399 | Laser vaporization, other than face, three or more lesions |
| 0400 | Cautery (electro, chemo) destruction or simple surgical excision of benign or premalignant lesions |
| 0401 | Cautery (electro, chemo, or simple surgical excision, one lesion) elsewhere |
| 0402 | Warts & fibrocutanous tags - simple |
| 0404 | Cryocautery, etc., of benign lesion of skin, etc., second lesion |
| 0405 | Cryocautery, etc., of benign lesion of skin, etc., subsequent lesions (each) |
| 0406 | Cryocautery, etc., of benign lesion of skin, etc., complicated lesions |
| 3300 | Rectum, villous papilloma of rectum, extensive, local excision |
| 3301 | Rectum, unlisted or unusually complicated |
| 3311 | Rectum, proctosigmoidoscopy |
| 3315 | Rectum, proctosigmoidoscopy with removal of polyp or papilloma, single |
| 3317 | Rectum, proctosigmoidoscopy with removal of polyp or papilloma, multiple |
| 3429 | Anus, unlisted or unusually complicated |
| 3994 | Urethroscopy, therapeutic, polyps, urethral, excision of fulguration with or without urethroscopy |
| 4000 | Urethra, urethroscopy, diagnostic, initial or subsequent |
| 4120 | Penis, unlisted or unusually complicated |
| 4221 | Scrotum, skin lesion, local excision |
| 4229 | Scrotum, unlisted or unusually complicated |
| 4611 | Cervix, local excision of lesion, cauterization of biopsy, one or more sites |
| 4641 | Cryosurgery of the cervix for other conditions |
| 8470 | General practice visit – regional gynaecological exam – including cytological smear - cervix |
| 8471 | General practice visit – regional gynaecological exam – no cytological smear |
| 8495 | Obstetrics / gynaecology visit – complete gynaecological exam – including cytological smear - cervix |
| 8496 | Obstetrics / gynaecology visit – regional gynaecological exam – including cytological smear - cervix |
| 8497 | Obstetrics / gynaecology visit – regional gynaecological exam – no cytological smear |
| 8498 | General practice visit – complete gynaecological exam – including cytological smear - cervix |
| 8499 | General practice visit – complete gynaecological exam – no cytological smear |
| 8501 | Office visits, regional, history and examination |
| 8502 | Office visits, complete or extensive re-examination for same illness |
| 8507 | Office visits, subsequent visit |
| 8509 | Office visits, regional or subsequent visit or well baby care |
| 8529 | Office visits, regional intermediate visit or subsequent visit or well baby care |

^*^ Only included if the ICD-9 diagnosis code was 078 and if an anogenital wart tariff claim followed it within two weeks.
